# Effects of Telerehabilitation on Gross Motor Function in Children with Cerebral Palsy: A Systematic Review

**DOI:** 10.3390/healthcare14070942

**Published:** 2026-04-03

**Authors:** Olga Maia, Daniel Moreira Gonçalves, Rui Vilarinho

**Affiliations:** 1 Associação do Porto de Paralisia Cerebral (APPC), 4200-253 Porto, Portugal; 2 FP-I3ID, BHS, Escola Superior de Saúde Fernando Pessoa, 4200-256 Porto, Portugal; rvilarinho@ufp.edu.pt; 3 Research Center in Physical Activity, Health and Leisure (CIAFEL), Faculty of Sports, University of Porto (FADEUP), 4200-450 Porto, Portugal; danielmgon@fade.up.pt; 4 Laboratory for Integrative and Translational Research in Population Health (ITR), 4050-600 Porto, Portugal; 5 CIR, ESS, Polytechnic of Porto, Rua Dr. António Bernardino de Almeida nº 400, 4200-072 Porto, Portugal

**Keywords:** telerehabilitation, cerebral palsy, gross motor function

## Abstract

**Background/Objectives**: Telerehabilitation expands access to specialized neuropediatric physiotherapy for families facing barriers related to geography, work, or caregiving. This systematic review aimed to summarize the evidence regarding the effects of telerehabilitation on gross motor function (GMF) in children with cerebral palsy (CP). **Methods**: An electronic search was conducted in the following databases: PubMed, Web of Science, Embase, and the Cochrane Library; Google Scholar was consulted for additional literature. The search targeted randomized and non-randomized intervention studies evaluating the effects of telerehabilitation on GMF in children with CP at various levels of the Gross Motor Function Classification System (GMFCS), as well as related functional outcomes. The risk of bias in the included studies was assessed using the original Cochrane Collaboration risk of bias tool. The certainty of evidence was graded according to the GRADE framework. **Results**: Five studies involving 152 children were included, with CP aged 2.5 to 17 years. Telerehabilitation programs varied in duration, frequency, and type of intervention, as well as in caregiver involvement, comparator conditions, and outcome measures. The included studies suggested potential benefits in GMF and related functional outcomes; however, findings were heterogeneous, and superiority over comparison conditions was not consistently demonstrated. **Conclusions**: Although the reviewed studies suggest that telerehabilitation may be a feasible and potentially beneficial approach for children with CP, the limited number of studies and variability of interventions highlight the need for caution in interpreting these findings. Further high-quality studies with standardized outcome reporting are needed to clarify its contribution to GMF.

## 1. Introduction

Cerebral palsy (CP) is recognized as the most common motor disability in childhood, with an estimated prevalence rate of 1.77 per 1000 live births, according to the Surveillance of Cerebral Palsy in Europe (SCPE) in 2015 [1]. Characterized by disturbances in movement and posture development, CP results in limitations in functional activity due to non-progressive disorders that occur in the developing brain [2]. The SCPE classification currently divides CP into three main types: spastic, dyskinetic, and ataxic [3]. In line with the Gross Motor Function Classification System (GMFCS), cerebral palsy is assessed based on the severity of functional limitations and impairments, ranging from level I (least affected) to level V (most severe) [4].

Regardless of classification or severity, early physiotherapy intervention is important for minimizing children’s difficulties. A lack of such intervention can impair motor function and negatively affect children’s functionality [5]. Evidence shows that motor interventions conducted in the child’s environment with family involvement have significant effects on learning and development, with home and family seen as major contributors to these outcomes [5,6].

According to the World Confederation for Physical Therapy (2020), telerehabilitation refers to the use of information technologies to deliver healthcare and rehabilitation services in home and/or community contexts [7]. It may include remote assessment, follow-up, caregiver education, supervision of therapeutic activities, and monitoring of home-based intervention programs. Compared with conventional face-to-face physiotherapy, telerehabilitation may improve access to specialized care, continuity of treatment, flexibility of service delivery, and caregiver involvement, although it does not replace all aspects of in-person intervention, particularly when tactile assessment, manual guidance, and direct physical interaction remain clinically relevant. This approach has existed for some time, but there is limited evidence to support it, especially compared to conventional treatment. However, due to the urgent implementation of social distancing measures during the Coronavirus disease 2019 (COVID-19) pandemic, interest in telerehabilitation among professionals and healthcare institutions has increased [8].

Telerehabilitation offers the opportunity to intensify certain aspects of physiotherapy, particularly by enabling repetition of specific exercises, which enhances intervention outcomes [8]. It also allows physiotherapists to guide parents in implementing strategies during daily routines, respecting the child’s pace and promoting developmental gains. Moreover, it engages the entire family in the therapeutic process, contributing to their empowerment [9]. Some studies have reported potential benefits of telerehabilitation in children with various diagnoses, including CP, across a range of motor and functional outcomes. These studies report improvements in lower limb endurance, upper limb dexterity, performance in daily living activities, processing skills, and visual perception [10,11,12].

However, most of the existing studies focus on web-based games and virtual reality, which often limit their applicability to children with mild impairments on the GMFM scale and exclude younger age groups [10]. In addition, telerehabilitation may be less suitable in situations in which tactile assessment, manual guidance, and direct physical interaction are clinically important. Nevertheless, several barriers limit access to intervention, including living in rural areas, complex clinical situations, and economic or professional constraints. While telehealth does not eliminate the need for in-person visits, it improves access to specialized care [8].

Therefore, this systematic review aimed to identify and summarize the evidence regarding the effects of telerehabilitation on gross motor function (GMF) in children with CP, while also considering related functional outcomes reported in pediatric rehabilitation studies, such as gait performance, manual function, participation, goal attainment, quality of life, and well-being, when these are considered relevant to contextualize the functional impact of the intervention.

## 2. Materials and Methods

This study was conducted as a systematic review, in accordance with the Preferred Reporting Items for Systematic Reviews and Meta-Analyses (PRISMA) guidelines [13]. The review protocol was registered in the International Prospective Register of Systematic Reviews (PROSPERO, ID CRD42024497111).

### 2.1. Eligibility Criteria

This systematic review included randomized and non-randomized intervention studies evaluating the effects of telerehabilitation on GMF in children with CP under the age of 18 years. Only studies published in English, Portuguese, Spanish, or French were included. Articles in which the intervention did not meet the World Confederation for Physical Therapy definition of telerehabilitation [7]; studies that did not report outcomes for children with CP as a separate subgroup; and studies involving web-based games or virtual reality were excluded. Given the limited number of studies available in this field, both study designs were considered eligible for inclusion.

### 2.2. Information Sources and Search Strategy

The Population, Intervention, Comparison, and Outcome (PICO) strategy was used to define the research components: Participants (P), children with cerebral palsy; Intervention (I), telerehabilitation; Comparison (C), in-person intervention or no intervention; and Outcome (O), differences in gross motor function and related functional outcomes, including gait performance, manual function, participation, goal attainment, quality of life, and well-being.

An electronic search was carried out in the databases PubMed, Web of Science, Embase, and Cochrane Library during April and May 2025. The search expression used is presented in Appendix A.

Additionally, Google Scholar was consulted to identify relevant gray and published literature not indexed in the main databases. Two reviewers screened and extracted the data independently.

### 2.3. Selection Process and Data Collection

All references were exported to the EndNote 21 reference management software program, and duplicate entries were removed. Records were identified through database searches and reference list screening during the identification phase. Two independent reviewers screened titles and abstracts. Based on the eligibility criteria, irrelevant studies were excluded (screening phase). Then, full texts of potentially eligible studies were assessed (eligibility phase), and those that met all inclusion criteria were included in this review.

### 2.4. Data Items

The following data were extracted: study details (author and year), participant characteristics (sample size), study methodology, objectives, duration of the intervention, and outcomes.

### 2.5. Risk of Bias Assessment

The risk of bias in the included studies was assessed using the original Cochrane Collaboration risk of bias tool [14]. Although this tool was primarily developed for randomized studies, it was applied consistently across all included intervention studies to ensure a uniform assessment framework. The assessment comprised the following domains: selection bias, performance bias, detection bias, attrition bias, reporting bias, and other bias. Each domain was rated as low, high, or unclear risk of bias, according to the information reported in each study. Two reviewers performed this assessment independently, and any disagreements were resolved through discussion until consensus was reached.

## 3. Results

### 3.1. Study Selection

**Figure 1:**
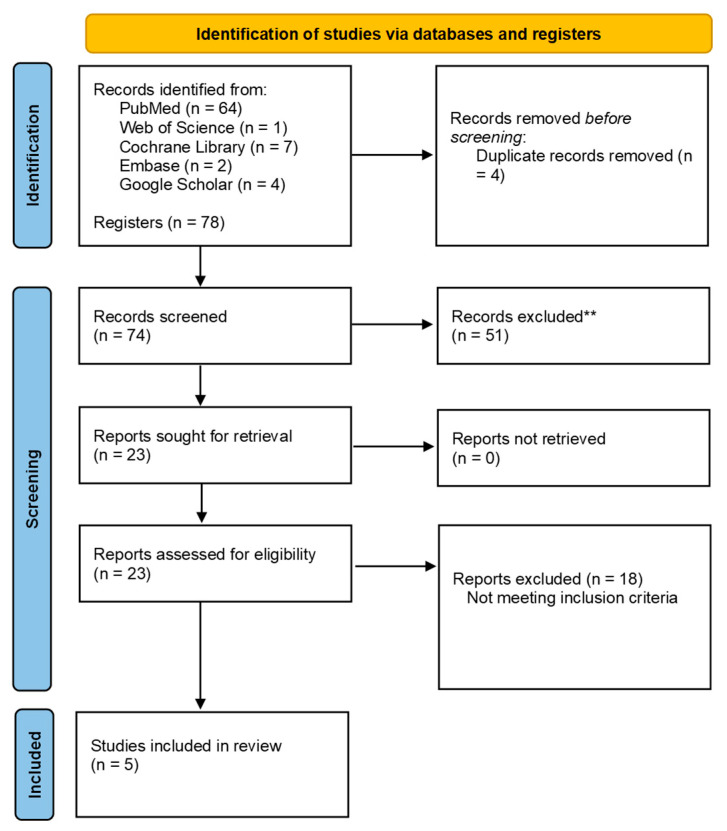
PRISMA flow diagram illustrating the study selection process, including identification, screening, eligibility assessment, and inclusion of studies in the systematic review. ** Records excluded for not meeting the inclusion criteria related to the research objective.

A total of 78 studies were initially identified through database searches and other sources. After removing duplicates, 74 records remained. During the screening process, 51 publications were excluded because they did not meet the eligibility criteria, and 18 were excluded for not being related to the research question. Five studies met the eligibility criteria and were included in this review (Figure 1).

### 3.2. Risk of Bias Assessment

A detailed assessment of the risk of bias in the included studies, using the original Cochrane Collaboration risk of bias tool, is presented in Table 1. Each domain was classified as having a low, high, or unclear risk.

### 3.3. Description of Included Studies

#### 3.3.1. Participants’ Characteristics

Five studies met the eligibility criteria and were included in this systematic review, which involved a total of 152 participants. Sample sizes ranged from 18 participants [18] to 43 participants [19]. Participants’ ages ranged from 2 years and 6 months [19] to 17 years [16].

#### 3.3.2. Intervention Protocols

The included studies demonstrate variability in the duration of programs and the frequency and length of sessions (Table 2).

In the study by Ferre et al. (2017), 24 children were divided into two groups of 12: the experimental group received Hand–Arm Bimanual Intensive Therapy (H-HABIT), and the control group received Lower Extremity Intensive Functional Training (LIFT) [17]. Both interventions lasted 9 weeks, with 2-hour sessions, 5 days a week, totaling 90 hours. The H-HABIT group performed caregiver-guided bimanual training focused on dexterity, manipulation, and assisted use of the affected hand through functional games and tasks. The LIFT group followed an equivalent intensity program targeting lower limb strength, balance, and coordination using playful tasks. Both groups continued their usual care. Caregivers received two in-person training sessions and weekly remote supervision via Adobe Connect, including video reviews and activity logs.

Surana et al. (2019) implemented a similar structure: the LIFT group performed 90 hours of lower limb intensive training over 9 weeks [15]. Activities were tailored to the functional level and included stair climbing, jumping, balance tasks, and obstacle negotiation. Caregivers selected daily exercises from a structured manual. The H-HABIT control group received equal training time but focused exclusively on structured bimanual upper limb tasks. Both groups received three caregiver training sessions, daily activity logs, and weekly remote supervision via Adobe Connect.

Sarti et al. (2021) conducted a different type of study, exploring the experience of telerehabilitation and well-being during the coronavirus disease 2019 (COVID-19) pandemic [18]. Of 56 participants, 18 children with cerebral palsy (CP) were separated into two groups: 9 received telerehabilitation (one session/week for 13 weeks), and 9 received no intervention. For those with motor needs, parents were sent instructional videos twice a week, and children participated in a weekly synchronous session with a therapist to monitor progress.

In the study by Celikel et al. (2023), both experimental and control groups followed the same motor-learning-based exercise protocol, three times per week for 40 min over eight weeks (24 sessions total) [16]. Activities included active stretching, stair climbing, walking on variable surfaces, functional object tasks, and sensory-motor challenges. Tasks were adapted and progressively intensified. In the telerehabilitation group, caregivers conducted exercises at home with support from pre-recorded videos and weekly remote supervision via Adobe Connect. Caregivers received training in motor learning principles and implementation strategies.

Finally, Sel et al. (2023) used a structured, goal-oriented home rehabilitation program supported by telerehabilitation [19]. The experimental group received one weekly online session with a physiotherapist over 12 weeks and practiced 40 minutes daily at home (56 additional hours). Caregivers underwent in-person training based on individual goals identified using the Canadian Occupational Performance Measure (COPM) and Goal Attainment Scaling (GAS). The control group received conventional care: two weekly in-person sessions at specialized centers focusing on physical functions such as stretching and orthotic use.

#### 3.3.3. Outcome Measures and Results

Ferre et al. (2017) hypothesized that H-HABIT would improve dexterity, bimanual manual function, and goal achievement [17]. The Box and Block Test (BBT) and Assistive Hand Assessment (AHA) were used for motor outcomes, while the COPM assessed caregiver perception. Results showed significant improvement in dexterity and functional goal performance, but no significant change in bimanual manual function. Caregivers received home activity instructions via telerehabilitation, including board games, clay modeling, and page-turning tasks. No significant differences were found between therapist and caregiver baseline assessments.

Surana et al. (2019) used the 1-Minute Walk Test (1MWT) as the primary outcome, and ABILOCO-Kids, the 30-Second Chair Rise Test, and single-leg stance as secondary outcomes [15]. Results indicated that lower limb intensive training at home led by caregivers significantly improved gait capacity and performance. Telerehabilitation using the LIFT model, supported by weekly remote supervision, increased walking distance and general ambulation ability.

Sarti et al. (2021) used two questionnaires to assess well-being: the Comprehensive Inventory of Thriving (CIT) and the Scale of Positive and Negative Experience (SPANE) [18]. Children with CP, regardless of telerehabilitation, showed higher scores in the “relationship” domain compared to typically developing children. The telerehabilitation group also reported higher scores in the “learning” dimension. However, there were no significant differences in perceived positive or negative emotions.

Celikel et al. (2023) assessed health-related quality of life using the Pediatric Quality of Life Inventory (PedsQL) 3.0 CP Module, a validated instrument for the Turkish population [16]. It includes 35 items across dimensions such as daily activities, balance, movement, pain, fatigue, school, feeding, and verbal communication. Self-reports were completed by children according to age-specific versions. Children in the telerehabilitation group showed significant improvements in pain, fatigue, feeding, and communication compared to the in-person group.

Sel et al. (2023) evaluated functional classification using four instruments: Gross Motor Function Measure-66 (GMFM-66): measures gross motor function, assessed via video by blinded evaluators; Pediatric Evaluation of Disability Inventory (PEDI): evaluates functional skills, caregiver assistance, and participation in daily activities; GAS: measures achievement of individual goals on a −2 to +2 scale, with ≥50 points indicating success; and COPM: assesses parent-perceived performance and satisfaction in meaningful activities [19]. These authors reported statistically significant gains across all instruments in the experimental group, with sustained effects at 24-week follow-up.

### 3.4. GRADE

As presented in Table 3, the certainty of evidence across outcomes was rated as low to moderate, reflecting a combination of methodological and contextual limitations.

For gross motor function and walking-related outcomes, the included studies generally reported favorable results. However, the evidence was downgraded due to small sample sizes, wide or unreported confidence intervals, and risk of bias related to home-based implementation and caregiver-led delivery, which limit the generalizability of the findings.

Regarding manual and bimanual function, health-related quality of life, and well-being, the certainty of evidence was judged to be generally low. This was primarily attributed to the reliance on single studies for each outcome, methodological limitations, and issues of indirectness, particularly in outcomes assessing well-being, which are not directly linked to gross motor function.

## 4. Discussion

The analysis of the five studies included in this review suggests that telerehabilitation may represent a feasible and potentially beneficial intervention approach for children with CP, particularly in contexts where access to in-person services is limited. However, the current evidence should be interpreted with caution, as the positive findings do not necessarily establish superiority over conventional face-to-face rehabilitation. Rather, the available studies indicate that telerehabilitation can support meaningful gains in selected domains, while also highlighting important methodological and contextual limitations that affect the strength and generalizability of the conclusions.

From a methodological perspective, four of the included studies used randomized or controlled experimental designs, which strengthen the internal validity of their findings [15,16,17,19]. Nonetheless, the overall body of evidence remains limited by the small number of available studies, modest sample sizes, and considerable heterogeneity across intervention protocols. Differences were observed not only in treatment duration and intensity, but also in therapeutic focus, caregiver involvement, delivery format, and outcome measures. This variability makes direct comparison difficult and limits the extent to which findings can be generalized across different clinical populations and rehabilitation settings. In contrast, Sarti et al. (2021) [18], although less rigorous in terms of experimental control, offer an original contribution by examining children’s subjective perceptions of well-being, thereby expanding the understanding of outcomes beyond strictly functional indicators.

A particularly important finding emerging from the review is the central role of caregivers in the implementation of telerehabilitation programs. Across the included studies, intervention delivery in the home environment depended substantially on caregiver participation, whether through supervision, facilitation of exercises, or communication with therapists. This family-centred model appears to be both feasible and clinically relevant, especially when combined with structured remote guidance from professionals. Cheung et al. [20] and Novak [5] support this perspective, arguing that home-based interventions may allow greater therapy intensity and better integration into everyday routines. Similarly, Camden et al. [21] suggest that telerehabilitation may promote parental empowerment by encouraging active collaboration between families and clinicians and by embedding therapeutic activities within the child’s natural environment.

At the same time, the apparent success of this model should not obscure the demands it places on families. None of the included studies systematically assessed caregiver training, confidence, burden, or empowerment, despite the likelihood that these factors directly influence adherence and treatment outcomes. This omission is clinically significant, as telerehabilitation may be less accessible or less effective in families facing low digital literacy, limited time availability, socioeconomic disadvantage, or high caregiving burden. Therefore, the feasibility reported in the included studies may reflect, at least in part, the characteristics of the families who were able to participate, rather than the universal applicability of telerehabilitation across all contexts. This issue reinforces the need for a more inclusive and equitable perspective when evaluating remote rehabilitation models [21].

Another notable aspect of the included studies is the diversity of outcomes assessed. Celikel, Sel, Surana, and Ferre primarily focused on objective clinical outcomes, such as gross motor function, bimanual performance, and goal achievement, whereas Sarti et al. [18] examined more subjective and ecological dimensions, including perceived support, respect, and learning. This diversity is valuable because it reflects the multidimensional nature of rehabilitation in children with CP. Telerehabilitation may influence not only physical performance, but also psychosocial well-being, engagement, and the child’s experience of participation in therapy. Previous literature has similarly suggested that remote rehabilitation can positively affect psychological well-being and cooperation during physical activities [22]. However, the heterogeneity of the outcome measures also complicates the synthesis of findings, since not all studies were evaluating the same constructs or targeting the same clinical domains.

Although the overall trend across studies was positive, some findings were more mixed. Ferre et al. [17], for example, reported no significant improvement in bimanual function as measured by the Assisting Hand Assessment (AHA), while Sarti et al. [18] did not identify significant differences in positive or negative effect between groups. These results are important because they suggest that the impact of telerehabilitation is not uniform across all domains of functioning and well-being. Instead, the effectiveness of telerehabilitation appears to depend on multiple interacting factors, including the therapeutic target, the intensity and structure of the intervention, the outcome measures selected, and the characteristics of the child and family.

Importantly, the studies included in this review do not provide sufficient evidence to conclude that telerehabilitation is more effective than conventional physiotherapy. In most cases, the evidence supports the view that telerehabilitation may serve as a complementary or alternative service-delivery model, rather than a replacement for in-person intervention. Its main advantages appear to include improving access to care, maintaining continuity of rehabilitation, and facilitating the incorporation of therapeutic activities into the home environment [5,20,21]. These potential benefits are especially relevant in situations where face-to-face therapy is disrupted or difficult to access. Accordingly, telerehabilitation should currently be understood as a flexible and potentially valuable rehabilitation strategy; however, its superiority over conventional physiotherapy cannot yet be established based on the available evidence.

With regard to the sustainability of outcomes, only Sel et al. [19] included a follow-up assessment, showing that the observed gains were maintained three months after the end of the intervention. This represents an important contribution, but it also highlights a major gap in the literature. The absence of longer-term follow-up in the remaining studies limits understanding of whether the reported improvements persist over time, generalize to daily life, or translate into meaningful developmental gains. In pediatric rehabilitation, this is a particularly relevant limitation, since the long-term maintenance and functional transfer of treatment effects are central to clinical relevance.

Finally, despite the clinical interest of the available studies, most were conducted with relatively small samples and in geographically restricted settings, namely Turkey, Italy, and the United States. This raises important questions about external validity, especially given potential differences in health systems, access to digital resources, family support structures, and cultural attitudes toward home-based care. Future research should therefore aim to include larger and more diverse samples, broader cultural contexts, and clearer characterization of participants, including CP subtype, severity level, age, and caregiver-related factors. Greater consistency in intervention reporting and outcome selection would also improve comparability across studies and strengthen the evidence base.

Overall, the findings of this review are consistent with previous studies reporting positive effects of telerehabilitation on motor, cognitive, and functional domains [23,24,25]. Nevertheless, the present review suggests that the current evidence is more supportive of feasibility and preliminary benefit than of definitive efficacy. Telerehabilitation appears to hold promise as a clinically useful and accessible model of care for children with CP, but further high-quality research is needed before firm conclusions can be drawn regarding its comparative effectiveness and long-term impact.

### Limitations

The most relevant limitations of the studies analyzed are: (1) the lack of follow-up evaluations in four out of the five studies, which prevents an in-depth analysis of the durability of intervention effects; (2) the considerable heterogeneity in frequency, duration, and structure of telerehabilitation programs, as well as in comparator conditions and outcome measures, which hinders direct comparisons and generalization of results; and (3) the absence of systematic evaluation of caregiver empowerment and training, despite its likely influence on adherence and treatment gains.

## 5. Conclusions

This review suggests that telerehabilitation may represent a feasible and potentially beneficial approach to intervention in children with CP, particularly in contexts where in-person therapy is limited. Positive findings were observed in indicators such as gross motor function, bimanual performance, achievement of therapeutic goals, and subjective well-being, although not all domains showed statistically significant changes, and conclusions specifically regarding gross motor function should be interpreted with caution, given the heterogeneity of the outcomes assessed across the included studies.

However, the overall certainty of evidence, as assessed by the GRADE framework, was rated as low to moderate due to methodological limitations, small sample sizes, heterogeneity in intervention protocols, and reliance on subjective measures in some studies. Standardizing intervention protocols, systematically assessing parental empowerment, and developing longitudinal studies are priorities for future research in this field.

Further high-quality studies, particularly randomized controlled trials with more standardized outcome reporting, are needed to clarify the specific contribution of telerehabilitation to gross motor function in children with cerebral palsy.

## Figures and Tables

**Table 1:** Risk of bias assessment of the included studies according to the original Cochrane Collaboration tool.

Author (Year)	Selection Bias	Performance Bias	Detection Bias	Attrition Bias	Reporting Bias	Other Bias
Surana et al. (2019) [15]	Low risk	Low risk	High risk	Low risk	High risk	Unclear risk
Celikel et al. (2023) [16]	Low risk	Low risk	High risk	Low risk	High risk	Unclear risk
Ferre et al. (2017) [17]	High risk	Low risk	High risk	Low risk	High risk	Unclear risk
Sarti et al. (2021) [18]	Unclear risk	Low risk	High risk	Low risk	Low risk	Unclear risk
Sel et al. (2023) [19]	High risk	High risk	High risk	High risk	High risk	Unclear risk

Note: Selection bias refers to random sequence generation and allocation concealment; performance bias refers to blinding of participants and personnel; detection bias refers to blinding of outcome assessment; attrition bias refers to incomplete outcome data; reporting bias refers to selective reporting; and other bias refers to additional sources of bias not covered by the previous domains.

**Table 2:** Summary of the included studies.

Author (Year)	Study Design/Objective	Sample	Intervention Protocol	Results
Sarti et al. (2021) [18]	RCT: Compare the impact of telerehabilitation on the well-being experience of children with special needs, compared to those who did not receive telerehabilitation and to typically developing children.	18 children with cerebral palsy (ages 7–14). Mixed CP profiles, including tetraplegia, hemiplegia, and diplegia. Functional levels ranged from mild to moderate–severe impairment according to VFCS, GMFCS, and MACS classifications. EG: 9 children with telerehabilitation; CG: 9 children without telerehabilitation (mean age: 131.37 months, SD 24.16).	Intensive individualized telerehabilitation program (real-time video sessions).	Significant differences were observed in the relational dimension among children with CP between the telerehabilitation and control groups, and in comparison to the typically developing group. No significant inter- or intra-group differences were found regarding perception of negative or positive feelings.
Surana et al. (2019) [15]	RCT: Compare the effectiveness of intensive lower limb functional training (LIFT) vs. upper limb bimanual training (H-HABIT).	24 children with unilateral spastic cerebral palsy (ages 3–13). All participants were classified as GMFCS levels I–II 12 in the LIFT group; 12 in the H-HABIT group.	HABIT and LIFT.	No significant differences in gait speed between groups (*p* = 0.39). Significant improvements in gait capacity and performance in the LIFT group (*p* = 0.008). No significant improvement in manual function in the H-HABIT group.
Ferre et al. (2017) [17]	RCT: Assess whether intensive bimanual hand–arm intervention improves dexterity, manual function, and goal achievement more than intensive lower limb functional intervention.	24 children with unilateral spastic cerebral palsy (ages 2.5–10.1 years). Functional classification (e.g., GMFCS or MACS levels) was not clearly reported. EG (H-HABIT): 12 children (mean age 5.2 ± 2.7); CG (LIFT): 12 children (mean age 5.8 ± 2.3).	HABIT and LIFT were remotely supervised.	The EG showed significant improvement in dexterity compared to the CG (*p* < 0.05). No significant differences in bimanual manual function (*p* > 0.05). EG showed significant improvement in caregiver-perceived performance (*p* < 0.05).
Celikel et al. (2023) [16]	RCT: Assess whether telerehabilitation based on motor learning principles was equivalent to in-person therapy in improving quality of life in children with CP during COVID-19.	25 children with spastic cerebral palsy (ages 3–17). GMFCS levels I–II. EG: 11 children in telerehabilitation (mean age 8.59 ± 4.69); CG: 14 children in in-person therapy (mean age 11.64 ± 4.83).	Video-supervised home exercise program of motor learning-based activities, 3 sessions/week, 8 weeks, total 24 sessions. Each session: 45 minutes.	Significant improvements in play activities, pain, fatigue, feeding, and speech-language communication (*p* < 0.05) post-treatment.
Sel et al. 2023 [19]	RCT: Evaluate the effectiveness of a telerehabilitation program for preschool-aged children with CP compared to an equivalent in-person program.	43 children (ages 3–6; mean age 4.66 ± 1.08) Functional classification was reported across GMFCS, MACS, and CFCS levels I–V, indicating a wide range of motor and communication abilities. EG: 23 children in telerehabilitation (mean age 4.63 ± 1.06); CG: 20 children in usual care (mean age 4.70 ± 1.14)	Home-based physiotherapy program delivered via videoconferencing; caregiver-guided. CG: 12 weeks, 2 in-person sessions/week (40 min) + usual care. EG: 12 weeks, daily 40-minute structured program + 1 hour/week telerehabilitation.	Both groups showed statistically significant pre- to post-test improvements in GMFM, COPM, GAS, and PEDI scores (*p* < 0.001). The experimental group additionally demonstrated significant gains in activity, participation, and goal attainment (*p* < 0.001).

Abbreviations: CP, cerebral palsy; RCT, randomized controlled trial; EG, experimental group; CG, control group; SD, standard deviation; VFCS, Viking Functional Communication Scale; GMFCS, Gross Motor Function Classification System; MACS, Manual Ability Classification System; CFCS, Communication Function Classification System; LIFT, Lower Extremity Intensive Functional Training; H-HABIT, Hand–Arm Bimanual Intensive Therapy Including Lower Extremities; GMFM, Gross Motor Function Measure; COPM, Canadian Occupational Performance Measure; GAS, Goal Attainment Scaling; PEDI, Pediatric Evaluation of Disability Inventory.

**Table 3:** Summary of findings (GRADE): telerehabilitation in children with cerebral palsy.

Study	Outcome	Participants/Follow-Up	Study Design	Certainty of Evidence (GRADE)	Relative Effect (95% CI)	Comments	Anticipated Absolute Effects
Sarti et al. (2021) [18]	Well-being (CIT/SPANE)	*n* = 18 (CP subsample); 13 weeks	Intervention study	LOW ⊕⊕◯◯	Not estimable	Improvements were reported in relational and learning dimensions, whereas no significant effects were found for positive or negative feelings. Downgraded for imprecision and indirectness.	Not estimable
Surana et al. (2019) [15]	Gait capacity/performance (1MWT; ABILOCO-Kids; strength/balance tests)	*n* = 24; 9 weeks	Intervention study	LOW ⊕⊕◯◯	Not estimable	Within-group improvements were observed in the LIFT group, but between-group differences were not consistently demonstrated across all gait-related measures. Downgraded for imprecision and risk of bias.	Not estimable
Ferre et al. (2017) [17]	Dexterity/manual function (BBT, AHA) and goal performance (COPM)	*n* = 24; 9 weeks + weekly telerehabilitation	Intervention study	LOW ⊕⊕◯◯	Not estimable	Improvements were reported in dexterity and caregiver-perceived performance, although no significant between-group differences were found across all manual outcomes. Downgraded for imprecision and risk of bias.	Not estimable
Celikel et al. (2023) [16]	Health-related quality of life (PedsQL 3.0 CP)	*n* = 25; 8 weeks	Intervention study	LOW ⊕⊕◯◯	Not estimable	Improvements were reported in selected quality-of-life domains. Downgraded for imprecision and risk of bias related to subjective outcome assessment.	Not estimable
Sel et al. (2023) [19]	Gross motor function and participation (GMFM-66, PEDI, COPM, GAS)	*n* = 43; 12 weeks (+24-week follow-up)	Intervention study	MODERATE ⊕⊕⊕◯	Not estimable	Significant gains were reported in GMFM, PEDI, COPM, and GAS outcomes, with effects maintained at follow-up. Downgraded for imprecision.	Not estimable

GRADE Working group grades of evidence: ⊕⊕⊕⊕ High quality: Further research is very unlikely to change our confidence in the estimate of effect. ⊕⊕⊕◯ Moderate quality: Further research is likely to have an important impact on our confidence in the estimate of effect and may change the estimate. ⊕⊕◯◯ Low quality: Further research is very likely to have an important impact on our confidence in the estimate of effect and is likely to change the estimate. ⊕◯◯◯ Very low quality: We are very uncertain about the estimate. Note: Certainty of evidence was downgraded mainly due to imprecision (e.g., small sample sizes, wide confidence intervals, or limited reporting of effect estimates), risk of bias related to lack of blinding and/or subjective or caregiver-reported outcome measures, and, where applicable, indirectness (e.g., CP subsamples or outcomes not directly aligned with gross motor function). “Not estimable” indicates that effect sizes or anticipated absolute effects could not be calculated from the available data. Abbreviations: CIT, Comprehensive Inventory of Thriving; SPANE, Scale of Positive and Negative Experience; CP, cerebral palsy; 1MWT, 1-Minute Walk Test; BBT, Box and Block Test; AHA, Assisting Hand Assessment; COPM, Canadian Occupational Performance Measure; PedsQL 3.0 CP, Pediatric Quality of Life Inventory 3.0 Cerebral Palsy Module; GMFM-66, Gross Motor Function Measure-66; PEDI, Pediatric Evaluation of Disability Inventory; GAS, Goal Attainment Scaling.

## Data Availability

No new data were created or analyzed in this study. Data sharing is not applicable to this article.

## Abbreviations

The following abbreviations are used in this manuscript:

CP	Cerebral Palsy
SCPE	Surveillance of Cerebral Palsy in Europe
GMF	Gross Motor Function
GMFCS	Gross Motor Function Classification System
RCT	Randomized Controlled Trial
LIFT	Lower Extremity Intensive Functional Training
H-HABIT	Hand–Arm Bimanual Intensive Therapy Including Lower Extremities
EG	Experimental Group
CG	Control Group
COPM	Canadian Occupational Performance Measure
GAS	Goal Attainment Scaling
PEDI	Pediatric Evaluation of Disability Inventory
BBT	Box and Block Test
CIT	Comprehensive Inventory of Thriving
SPANE	Scale of Positive and Negative Experience
PedsQL	Pediatric Quality of Life Inventory
1MWT	1-Minute Walk Test
AHA	Assisting Hand Assessment
GMFM-66	Gross Motor Function Measure-66 items

## Appendix A

**Table A1:** Search strategies used in the selected databases.

Web of Science:
Title ((Telerehabilitation OR Telemedicine OR “home-based intervention”) AND (“Cerebral palsy”)) AND (pediatric OR childhood OR children) AND Abstract ((Telerehabilitation OR Telemedicine OR “home-based intervention”) AND (“Cerebral palsy”)) AND (pediatric OR childhood OR children)
PubMed:
((Telerehabilitation [Title/Abstract] OR Telemedicine [Title/Abstract] OR “home-based intervention” [Title/Abstract]) AND (“Cerebral palsy” [Title/Abstract])) AND (pediatric [Title/Abstract] OR childhood [Title/Abstract] OR children [Title/Abstract])
Embase:
((Telerehabilitation OR Telemedicine OR “home-based intervention”) AND (“Cerebral palsy”) AND (pediatric OR childhood OR children))
Cochrane Library:
((Telerehabilitation OR Telemedicine OR “home-based intervention”) AND (“Cerebral palsy”) AND (pediatric OR childhood OR children))
Google Scholar:
Search conducted using keywords: “telerehabilitation”, “cerebral palsy”, “children”. Used as a supplementary source.
